# A Comparison of Apical Transportation in Severely Curved Canals Induced by Reciproc and BioRaCe Systems 

**Published:** 2014-03-08

**Authors:** Mohammadreza Nabavizadeh, Abbas Abbaszadegan, Leila Khojastepour, Mohsen Amirhosseini, Ebrahim Kiani

**Affiliations:** a * Department of Endodontics, Dental School, Shiraz University of Medical Sciences, Shiraz, Iran; *; b * Department of Radiology, Dental School, Shiraz University Medical Sciences, Shiraz, Iran; *; c * Private Practice, Shiraz, Iran*

**Keywords:** Apical Transportation, BioRaCe, Endodontic, Nickel-Titanium Alloy, Reciproc, Root Canal Preparation, Rotary Files

## Abstract

**Introduction:** Preserving the apical root structure during cleaning and shaping of the canal has always been a challenge in endodontics particularly when the root canals are curved. The purpose of this *in vitro* study was to compare the apical transportation induced by the Reciproc and BioRaCe rotary systems in preparing the mesiobuccal root canal of the human maxillary molars. **Materials and Methods:** The mesiobuccal canals of sixty extracted maxillary molars with curvature angle of 25˚-35˚ were selected and randomly assigned into two groups. Each canal was prepared by either Reciproc or BioRaCe rotary systems. A double-digital radiographic technique and AutoCAD software were used to compare the apical transportation at 0.5, 1, 2, 3, 4 and 5 mm distances from the working length (WL). The distance between the master apical rotary file and the initial K-file in the superimposed radiographs determined the amount of apical transportation. An independent t-test was used to compare the groups. The statistical significant level was set at 0.05. **Results:** Apical transportation of the Reciproc group was significantly greater than the BioRaCe group in all distances (*P*<0.001). The maximum apical transportation occurred in the Reciproc group at 0.5 mm from the WL (0.048±0.0028 mm) and the minimum occurred for BioRaCe at 5 mm from the WL (0.010±0.0005 mm). **Conclusions:** The Reciproc system produced significantly more apical transportation than the BioRaCe, but this fact does not seem to negatively alter the clinical success or quality of root canal treatment.

## Introduction

Cleaning and shaping of the root canal system is a critical phase in endodontic treatment [1]. During canal preparation with stainless steel hand instruments, deviation from the original shape of the canal might occur to some extend [2, 3]. These changes may have a negative impact on the quality of endodontic treatment by diminishing the efficiency of disinfection procedures and can possibly have an adverse effect on quality of obturation [4-6]. To defeat the drawbacks of stainless steel instruments, endodontic files made of nickel–titanium (NiTi) alloy were proposed [7].

With a special design, RaCe rotary system (FKG. Dentaire SA, La Chaux-de-Fonds, Switzerland), has been addressed by several studies to effectively clean and shape the root canal system while producing more centered canal shape [8-14]. Files of this system have a triangular cross-section design and alternating cutting edges [3, 11, 13]. The BioRaCe system, with same physical characteristics to RaCe, is presented to the market and is different from the regular RaCe instruments in size, taper, sequence and shank codes. The changes in sequence of sizes and tapers have allowed the required apical sizes to be achieved with fewer instruments [15]. The BioRaCe basic kit is consisted of six instruments naming BR0; 25/0.08, BR1; 15/0.05, BR2; 25/0.04, BR3; 25/0.06, BR4; 35/0.04 and BR5; 40/0.04. Depending on the root canal anatomy, the final apical preparation might be achieved by using few numbers of these instruments [16].

A new single-file system with reciprocal movement named Reciproc (VDW, Munich, Germany) is introduced to the market. The reciprocal motion is considered as the engine-driven counterpart of balanced-force hand technique [17], which was first introduced for hand preparation of severely curved canals [18]. Reciproc system is claimed to be capable of shaping the root canal systems thoroughly with only one instrument. Files of this system have been constructed by a new heat-treatment operation from a special alloy called M-Wire NiTi. These single-use files have an increased flexibility and more resistance to cyclic fatigue than traditional NiTi files [19, 20]. Moreover, the S-shaped cross-section design of these files has produced two effective cutting edges. Different size and various tapering of the Reciproc files are available as follows: R25; tip Size #25 with apical taper of 8% (25/0.08), R40; tip size #40 with an apical taper of 6% (40/0.06), R50; tip size #50 with an apical taper of 5% (50/0.05) [17]. A special handpiece is introduced for application of these instruments with a reciprocal motion [17]. With the benefit of these useful features, this system can also be used in the curved canals.

Measuring the apical transportation may be carried out by different techniques but it can be problematic as each technique has its own limitations and there is no relating gold standard method for it [21]. The double radiographic super imposition technique proposed by Iqbal *et al.* is one of the most efficient, easy-to-use and cost-effective methods which can determine the maximum real curvature of the canal [22]. This method enables the evaluation of the radiographs taken before and after root canal preparation to detect the probable aberration(s) from the original shape of the canal.

To the best of our knowledge, no study exists in the literature comparing the apical transportation of the Reciproc and BioRaCe systems. Hence, this *in vitro* study was set up to measure and compare the apical transportation of these two rotary systems during the preparation of the mesiobuccal roots of the extracted human maxillary molars.

## Methods and Materials

This study was approved by the Ethics Committee of Shiraz University of Medical Sciences (Grant no. 3644-03-01-90). Sixty extracted maxillary molars with mesiobuccal root curvature within the range of 25-35˚ and the radii of curvature between 3.5 to 10 mm, calculated according to methodology of Schneider [23] and Pruett *et al.* [24], were selected among 303 teeth that were extracted due to the extensive caries and the periodontal problems. The teeth were disinfected with 0.5% sodium hypochlorite (NaOCl), to become free of any tissue fragments or calcified debris and were double-checked for any defects and flaws. The presence of second mesiobuccal canal was assessed using stereomicroscope (Carl Zeiss AG, Oberkochen, Germany) at 40× magnification and was a criterion for tooth exclusion. The selected teeth were then stored in a 10% formalin solution. Access cavities were prepared using a #4 round diamond bur (SS White Burs, Lakewood Inc, USA) in a high-speed handpiece under copious irrigation. To determine the working length (WL) a #15 K-file (Dentsply Maillefer, Ballaigues, Switzerland) was inserted into the canal until it became visible at the apical foramen under the stereomicroscope. The WL was calculated to be 1 mm less than the length attained by the initial appearance of the file. The canals in which the #15 K-file was not bound in to the apical constriction were excluded. Furthermore, roots with dissimilar lengths were also excluded from this experiment. Each tooth was embedded in an acrylic resin block and attached to a goniometer turntable which was fixed on a platform. 

A Plexiglas jig was designed for confident standardization of the experimental condition, so that the repeatable positions of the x-ray cone and the sensor would be assured during the study for each sample. The double–digital radiographic technique was used to compare the apical transportation in the same way described by Iqbal *et al.* [22].

A #15 K-file was inserted in the root canal to the WL and several radiographic images were obtained as the turntable was gradually rotated. When the file in the root canal appeared straight in a radiographic view, the turntable was turned 90 degrees where the maximum curvature of the root canal was visible. This view was considered as a baseline radiograph and the settings were recorded as an index for the following radiographs. This procedure was repeated for all samples.

The degree of root curvature and the radius of curvature of the central axis of the K-file were determined by AutoCAD 2010 (Autodesk, San Rafael, CA, USA). To ensure the standardization, the radius of curvature and curvature angles of the selected root canals for each group were assessed. Teeth with completely formed roots and matching the aforementioned criteria were selected and included in this study. The selected samples were randomly assigned into two experimental groups.

Instruments were set in a 6:1 contra-angle handpiece (Sirona Dental Systems GmbH, Bensheim, Germany) attached to an endodontic torque-limited electric motor (Silver; VDW GmbH, Munich, Germany) and all the procedures were performed by the same operator.

In Group A (*n*=30), the electric motor was adjusted to pre-programmed torque and speed settings for the BioRaCe files (a speed of 500 rpm and a maximum torque of 1 Ncm), then the samples were instrumented with BioRaCe files (FKG. Dentaire SA, La Chaux-de-Fonds, Switzerland), using the BR0 (25/0.08) to the BR3 (25/0.06) instrument according to the manufactures’ instructions by a gentle in-and-out motion.

**Figure 1:**
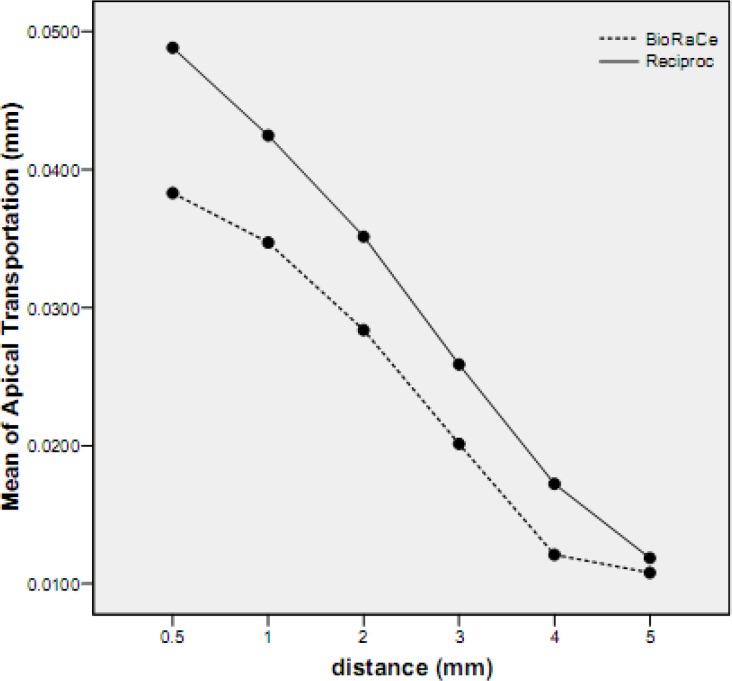
Mean apical transportation (mm) for the experimental groups

In Group B (*n*=30), the electric motor was adjusted to pre-programmed torque and speed settings for the Reciproc files (10 cycles of reciprocation per second, the equivalent of 300 rpm), then the samples were prepared with a R25 file (Reciproc 25/0.08, VDW, Munich, Germany) according to the manufacturers’ instructions by a light in-and-out pecking motion. The instruments were removed from the canal after each three pecks to clean the accumulated debris in flutes. In both groups, each instrument was considered as a single-use instrument.

In BioRaCe group, root canal irrigation was performed using 2 mL of 2.5% NaOCl between each instrument change. In Reciproc group, 2 mL of 2.5% NaOCl was used between each three file peckings. The tip of each file was covered with RC-Prep paste (Premier Dental Products, Philadelphia, USA) to facilitate proper instrumentation. After preparation was complete, the smear layer was removed by using 17% ethylenediamine-tetraacetic acid (EDTA) (Asia Chemi Teb Co., Tehran, Iran) and 2.5% NaOCl. A glide path was established with a #10 K-file before preparation of both groups. The patency was checked with a #08 K-file after using each instrument.

The prepared teeth were put on the constructed radiographic jig at the formerly recorded degree of rotation. A postoperative radiograph was taken with the master apical rotary file inserted into the canal to the working length.

All digital radiographic images were taken using the Digora PCT System (Soredex; Orion Corporation Ltd., Helsinki, Finland). The images were saved in JPG format and imported in to Adobe Photoshop CS4 (Adobe Systems Inc., San Jose, CA, USA) to enhance the edges of the pre-and post instrumentation radiographs. The images were then transferred to AutoCAD 2010 to superimpose the initial and final radiographs. An independent expert blindly measured the deviation from the initial K-file in pre-operative radiograph to the central axis of the master apical rotary file in post-operative radiograph at 0.5, 1, 2, 3, 4 and 5 mm distances from the apex. The mean and standard deviations were obtained for each group and the independent sample t-test was used to find statistically significant differences between the two groups. *P*<0.05 was considered statistically significant.

**Table 1:** Apical transportation (mm) at different distances from the working length (*P*<0.001)

**Distance ** **from apex **	**Mean (SD)**	
	**BioRaCe**	**Reciproc**
**0.5 mm **	0.038 (0.0028)	0.048 (0.0028)
**1 mm**	0.034 (0.0045)	0.042 (0.0028)
**2 mm**	0.028 (0.0030)	0.035 (0.0031)
**3 mm**	0.020 (0.0008)	0.025 (0.0030)
**4 mm**	0.012 (0.0009)	0.017 (0.0020)
**5 mm**	0.010 (0.0005)	0.011 (0.0040)

## Results

There was no incidence of instrument separation in any of the cases. No statistically significant differences were observed between the radii of curvature and curvature angles of the selected root canals for each group by employing the Student’s t-test. Both groups showed small deviation from the original shape of the canal. The apical transportation induced by the Reciproc files was significantly greater than the BioRaCe group in all distances (*P*<0.001). The maximum apical transportation occurred at 0.5 mm of the WL for Reciproc group (0.048±0.0028) and the minimum was at 5 mm from the WL for the BioRaCe group (0.010±0.0005). In both groups, the apical transportation decreased when the distance from the WL increased (Figure 1). The mean values of apical transportation for each group are summarized in Table 1.

## Discussion

One of the important purposes of the root canal preparation strategy is to form a gradually tapered emergence for the root canal while keeping its original shape [1]. However, some iatrogenic errors may occur during the shaping of narrow and curved canals. Examples of these mishaps are canal transportation, elbow or ledge formation and canal obstruction [25, 26].

By definition described in 2003 by the American Association of the Endodontics (AAE), apical transportation, ledge formation and perforations may take place by removal of the canal wall structure on the outside of canal curvature, owing to the propensity of the files to self-return to their original straight shape during preparation of the canal [27].

As stated by Weine, once transportation has occurred it is impossible to get back to the original canal shape, particularly in curved canals [28]. In other words, it may lead to an hourglass-shaped anatomy at the apical end of the canal and leaving infected pulp tissue on untouched walls. It also can lead to a ledge or perforation if improper instrumentation is continued [29].

Various studies have demonstrated that NiTi rotary instruments in comparison to stainless steel files, can better maintain the original shape of the canals [30-32]. Among rotary systems, several reports have shown that the RaCe rotary system is capable of keeping the original morphology of the root canal during instrumentation [8-13]. Until the time of this study, there are limited studies regarding the cleaning efficacy and the shaping ability of Reciproc rotary file system. In an assessment by Burklein *et al.*, the shaping ability of two single-file systems with reciprocal movements, *i.e.* Reciproc and WaveOne instruments, was compared to systems with rotational movements (Mtwo and ProTaper), in curved canals [33]. By determining the degree of straitening, they found that all tested instruments were safe and able to keep the original curvature of the root canal. Similar findings were confirmed again in another study by the same researchers [17]. Another survey which was performed in resin simulated curved canals by Yoo and Cho, revealed that Reciproc and WaveOne instruments had good shaping ability and could maintain the original canal curvature better than the ProTaper and Profile systems [34]. In the current study we evaluated the apical transportation of a reciprocating single-file system (*i.e.* Reciproc) with BioRaCe system during preparation of the mesiobuccal root of the extracted maxillary molars.

Several methods can be used to evaluate and compare the preparation of the root canals before and after instrumentation [35-38]. Radiographic imaging technique was selected for this study since no physical interfering was required. However, this method has some drawbacks like inability to observe three-dimensional view and cross-section of the root canals [39].

For the radiographic evaluation of the apical transportation, both the mesiodistal and buccolingual views can be used, albeit they might not be able to reveal the actual transportation. To overcome this issue and to present the utmost real curvature of the canal, we took several radiographic projections to obtain the most approximate outlook of the canal. The mesiobuccal roots of the extracted human maxillary molars were preferred for this study since they usually present with remarkable curvatures and have mesiodistal flattening [40]. Besides, we used similar teeth and root canals with same length and morphology in an attempt to make comparability of the experimental groups. Roots with second mesiobuccal canals were not used as they might have various anatomical configurations. To keep the condition of our study similar to clinical practice, the crowns of the teeth were not resected as during root canal preparation, they might imply pressure on the files [41].

In this study, extracted teeth were used instead of resin blocks. Resin materials have different mechanical properties compared to human dentin. As their hardness, compressive resistance and elasticity are lower than dentin, the preparation breakdowns such as transportation may occur with less frequency [42]. Moreover, the possible created heat during instrumentation in resin blocks may soften the resin materials which may glue to the cutting blades [43]. To pass over any possible effect of the air pressure on the torque and speed, an electric torque-controlled motor was employed instead of air-driven systems [44]. 

Although the reciprocal motion was introduced for preparation of severely curved canals [18], our results revealed that the Reciproc files significantly produced more transportation that BioRaCe group. This finding can be attributed to the superior flexibility of the RaCe files or might be ascribed by the presence of sharp cutting edges in Reciproc files. It is notable that the greater flexibility of the RaCe files can be credited to their special design and the segments on their working surface [19, 20].

In an investigation on shaping ability of RaCe rotary instruments in simulated root canals, Rangel *et al.* revealed that these instruments were able to rapidly prepare the simulated canals with little changes in WL and few aberrations in canal configuration [10]. These findings were in accordance with our results. Based on our findings, it may be speculated that the centered apical preparation of a root canal may depend on the file design and its flexibility or the instrumentation technique as also stated by Bergmans *et al.* [45].

Wu *et al.* demonstrated that more than 0.3 mm apical transportation will negatively affect the root canal seal [4]. In the present study, the maximum apical transportation induced by both Reciproc and BioRaCe systems was at 0.5 mm of the WL. Our findings indicated that the maximum apical transportations were 0.048 and 0.038 mm for Reciproc and BioRaCe systems, respectively. These results were lower than some previously published data reporting the apical transportation induced by other rotary systems [22, 46].

Further studies with different methodologies should be performed to investigate more on performance of endodontic instruments within the root canal and to assess the transportation of the new instruments and techniques.

## Conclusion

Under the condition of this *in vitro* study, both systems created a slight alteration in original shape of the canals. Although the Reciproc system produced significantly more apical transportation than the BioRaCe system, this might not be clinically noteworthy as it may not affect the quality of root canal treatment.

## References

[1] Schilder H (1974). Cleaning and shaping the root canal. Dent Clin North Am.

[2] Peters OA, Peters CI, Cohen S, Hargreaves KM (2006). Cleaning and shaping of the root canal system. Pathway of the pulp.

[3] Ehsani M, Zahedpasha S, Moghadamnia AA, Mirjani J (2011). Comparison of race and Mtwo nickel titanium (NITI) rotary instrument with stainless steel K-Flexofile hand instrument in root canal centering & transportation. Iran Endod J.

[4] Wu MK, Fan B, Wesselink PR (2000). Leakage along apical root fillings in curved root canals. Part I: effects of apical transportation on seal of root fillings. J Endod.

[5] Mantri SP, Kapur R, Gupta NA, Kapur CA (2012). Type III apical transportation of root canal. Contemp Clin Dent.

[6] Pettiette MT, Delano EO, Trope M (2001). Evaluation of success rate of endodontic treatment performed by students with stainless-steel K-files and nickel-titanium hand files. J Endod.

[7] Walia HM, Brantley WA, Gerstein H (1988). An initial investigation of the bending and torsional properties of Nitinol root canal files. J Endod.

[8] Paqué F, Musch U, Hülsmann M (2005). Comparison of root canal preparation using RaCe and ProTaper rotary Ni-Ti instruments. Int Endod J.

[9] Yoshimine Y, Ono M, Akamine A (2005). The shaping effects of three nickel-titanium rotary instruments in simulated S-shaped canals. J Endod.

[10] Rangel S, Cremonese R, Bryant S, Dummer PM (2005). Shaping ability of RaCe rotary Ni-Ti instruments in simulated root canals. J Endod.

[11] Javaheri HH, Javaheri GH (2007). A comparison of three Ni-Ti rotary instruments in apical transportation. J Endod.

[12] McSpadden JT (2007). Mastering endodontic instrumentation.

[13] Pasternak-Junior B, Sousa-Neto MD, Silva RG (2009). Canal transportation and centring ability of RaCe rotary instruments. Int Endod J.

[14] Azimi S, Delvari P, Hajarian HC, Saghiri MA, Karamifar K, Lotfi M (2011). Cyclic fatigue resistance and fractographic analysis of race and protaper rotary NiTi instruments. Iran Endod J.

[15] Bonaccorso A, Cantatore G, Condorelli GG, Schafer E, Tripi TR (2009). Shaping ability of four nickel-titanium rotary instruments in simulated S-shaped canals. J Endod.

[16] Debelian G, Trope M (2008). BioRaCe: efficient, safe and biological based sequence files. Roots.

[17] Burklein S, Benten S, Schafer E (2013). Shaping ability of different single-file systems in severely curved root canals of extracted teeth. Int Endod J.

[18] Roane JB, Sabala CL, Duncanson Jr MG (1985). The “balanced force” concept for instrumentation of curved canals. J Endod.

[19] De-Deus G, Brandao MC, Barino B, Di Giorgi K, Fidel RA, Luna AS (2010). Assessment of apically extruded debris produced by the single-file ProTaper F2 technique under reciprocating movement. Oral Surg Oral Med Oral Pathol Oral Radiol Endod.

[20] Varela-Patino P, Ibanez-Parraga A, Rivas-Mundina B, Cantatore G, Otero XL, Martin-Biedma B (2010). Alternating versus continuous rotation: a comparative study of the effect on instrument life. J Endod.

[21] Iqbal MK, Floratos S, Hsu YK, Karabucak B (2010). An in vitro comparison of Profile GT and GTX nickel-titanium rotary instruments in apical transportation and length control in mandibular molar. J Endod.

[22] Iqbal MK, Maggiore F, Suh B, Edwards KR, Kang J, Kim S (2003). Comparison of apical transportation in four Ni-Ti rotary instrumentation techniques. J Endod.

[23] Schneider SW (1971). A comparison of canal preparations in straight and curved root canals. Oral Surg Oral Med Oral Pathol.

[24] Pruett JP, Clement DJ, Carnes DL Jr (1997). Cyclic fatigue testing of nickel-titanium endodontic instruments. J Endod.

[25] Deplazes P, Peters O, Barbakow F (2001). Comparing apical preparations of root canals shaped by nickel-titanium rotary instruments and nickel-titanium hand instruments. J Endod.

[26] Hulsmann M, Schade M, Schafers F (2001). A comparative study of root canal preparation with HERO 642 and Quantec SC rotary Ni-Ti instruments. Int Endod J.

[27] American Association of Endodontists (2003). Glossary of Endodontic Terms.

[28] Weine FS (2006). Endodontic Therapy.

[29] Zuolo ML, Walton RE, Imura N (1992). Histologic evaluation of three endodontic instrument/preparation techniques. Endod Dent Traumatol.

[30] Glosson CR, Haller RH, Brent Dove S, del Rio CE (1995). A comparison of root canal preparations using Ni-Ti hand, Ni-Ti engine-driven, and K-Flex endodontic instruments. J Endod.

[31] Short JA, Morgan LA, Baumgartner JC (1997). A comparison of canal centering ability of four instrumentation techniques. J Endod.

[32] Park H (2001). A comparison of Greater Taper files, ProFiles, and stainless steel files to shape curved root canals. Oral Surg Oral Med Oral Pathol Oral Radiol Endod.

[33] Burklein S, Hinschitza K, Dammaschke T, Schafer E (2012). Shaping ability and cleaning effectiveness of two single-file systems in severely curved root canals of extracted teeth: Reciproc and WaveOne versus Mtwo and ProTaper. Int Endod J.

[34] Yoo YS, Cho YB (2012). A comparison of the shaping ability of reciprocating NiTi instruments in simulated curved canals. Restor Dent Endod.

[35] Hülsmann M, Peters OA, Dummer PMH (2005). Mechanical preparation of root canals: shaping goals, techniques and means. Endod Topics.

[36] Berutti E (1993). Computerized analysis of the instrumentation of the root canal system. J Endod.

[37] Nielsen RB, Alyassin AM, Peters DD, Carnes DL, Lancaster J (1995). Microcomputed tomography: an advanced system for detailed endodontic research. J Endod.

[38] Nagy CD, Bartha K, Bernath M, Verdes E, Szabo J (1997). A comparative study of seven instruments in shaping the root canal in vitro. Int Endod J.

[39] Dowker SE, Davis GR, Elliott JC (1997). X-ray microtomography: nondestructive three-dimensional imaging for in vitro endodontic studies. Oral Surg Oral Med Oral Pathol Oral Radiol Endod.

[40] Gani O, Visvisian C (1999). Apical canal diameter in the first upper molar at various ages. J Endod.

[41] Vanni JR, Santos R, Limongi O, Guerisoli DM, Capelli A, Pecora JD (2005). Influence of cervical preflaring on determination of apical file size in maxillary molars: SEM analysis. Braz Dent J.

[42] Bertrand MF, Lupi‐Pégurier L, Médioni E, Muller M, Bolla M (2001). Curved molar root canal preparations using HERO 642 rotary nickel–titanium instruments. Int Endod J.

[43] Kum KY, Spangberg L, Cha BY, Il-Young J, Msd, Seung-Jong L, Chan-Young L (2000). Shaping ability of three ProFile rotary instrumentation techniques in simulated resin root canals. J Endod.

[44] Limongi O, Klymus AO, Baratto Filho F, Vanni JR, Travassos R (2004). In vitro evaluation of the presence of apical deviation with employment of automated handpieces with continuous and alternate motion for root canal preparation. J Appl Oral Sci.

[45] Bergmans L, Van Cleynenbreugel J, Beullens M, Wevers M, Van Meerbeek B, Lambrechts P (2003). Progressive versus constant tapered shaft design using NiTi rotary instruments. Int Endod J.

[46] García M, Duran-Sindreu F, Mercadé M, Bueno R, Roig M (2012). A Comparison of Apical Transportation between ProFile and RaCe Rotary Instruments. J Endod.

